# Coal Calorific Value Prediction via Multi-View Transformer

**DOI:** 10.3390/s26134244

**Published:** 2026-07-04

**Authors:** Donglian Zhang, Junzhuang Li, Zhefei Tian, Yilu Guo, Xiaoqiang Ren, Wenqi Ren, Xiang Li, Peiyi Zhang

**Affiliations:** 1 National Environmental Protection Research Institute for Electric Power Co., Ltd., Nanjing 210031, China; 12062690@ceic.com (D.Z.); 12057505@ceic.com (J.L.); 12009787@ceic.com (X.L.); 20093503@ceic.com (P.Z.); 2 Guoneng Nanjing Coal Quality Supervision and Inspection Co., Ltd., Nanjing 210031, China; 3 Hikvision Research Institute, Hangzhou 310051, China; guoyilu5@hikvision.com (Y.G.); renxiaoqiang@hikvision.com (X.R.)

**Keywords:** NIRS-XRF fusion spectroscopy, coal quality analysis, calorific value prediction, deep learning, masked autoencoder, multi-view transformer

## Abstract

Accurate measurement of coal calorific value is critical for efficient power generation. To address the limitations of conventional models on large-scale, heterogeneous datasets, this study proposes a novel deep learning framework, the Multi-View Transformer (MVFormer), utilizing fused Near-Infrared Spectroscopy (NIRS) and X-ray Fluorescence (XRF) data. The architecture employs a dual-pathway Transformer with a Masked Autoencoder pre-training strategy to enhance feature representation from over 20,000 coal samples. Furthermore, a multi-view fusion mechanism integrates diverse pre-processing perspectives to enhance generalization. Experimental results demonstrate that this approach significantly outperforms traditional Partial Least Squares (PLS) regression and Multilayer Perceptron (MLP) models. These findings validate the framework as a robust and precise solution for real-time industrial coal quality analysis, successfully achieving precise prediction of calorific value on large-scale coal datasets.

## 1. Introduction

Coal is a vital fossil fuel for thermal power generation, and its safe and efficient use is increasingly important amid growing global environmental concerns [1,2,3]. Coal samples vary significantly in quality and composition, which directly influences their energy efficiency and environmental impact [4]. The calorific value reflects the heat released per unit mass of coal and is an important indicator of coal quality. Therefore, accurate real-time measurement of calorific value is essential for efficient energy utilization.

In China, coal preparation plants commonly follow the national standard GB/T 213-2008 [5] and use the oxygen bomb combustion method to determine calorific value [6]. However, this approach is time-consuming and involves multiple complex steps. Online measurement systems based on prompt gamma neutron activation analysis (PGNAA) are available but involve radioactive risks, high costs, and substantial maintenance [7,8]. Recently, spectral sensing techniques such as laser-induced breakdown spectroscopy (LIBS), Near-Infrared Spectroscopy (NIRS), and X-ray Fluorescence spectroscopy (XRF) have been applied to assess coal properties.

LIBS uses a high-energy laser to generate plasma from coal samples, producing characteristic emission spectra for analysis. It offers real-time, non-radiative detection but is sensitive to laser energy fluctuations and environmental conditions, which can affect measurement reproducibility [9,10,11]. NIRS is a non-destructive method well-suited for analyzing organic components in coal, particularly hydrogen-containing functional groups [12,13]. Meanwhile, XRF is effective for detecting inorganic ash-forming elements like Al, Si, Fe, and Ca by measuring the fluorescence emitted from excited atoms [14,15]. However, relying on a single spectroscopic technique may limit the completeness of the chemical information obtained. To address this, fusing heterogeneous spectral data has emerged as a powerful strategy to enrich information content and enhance predictive accuracy. Combinations such as LIBS-NIRS [16], LIBS-XRF [17,18], and NIRS-XRF [19,20] have been explored to leverage the synergy between different techniques. Considering that coal calorific value is influenced by both organic and inorganic compositions, this study employs fused NIRS-XRF spectroscopy. This combination effectively integrates the detection of organic functional groups with inorganic element analysis, providing a comprehensive basis for precise coal quality prediction.

Conventional machine learning methods such as Partial Least Squares (PLS) [9,14,19,21] and random forest (RF) [19,20,22] have been widely applied to model the relationship between spectral data and coal calorific value. These methods are well suited for scenarios involving limited sample sizes, narrow coal source ranges, and relatively consistent compositional patterns. However, when confronted with large-scale datasets spanning diverse coal origins, wide rank distributions, and highly heterogeneous chemical compositions, their modeling capacity becomes insufficient. In such cases, PLS often underfits due to its linear assumptions, while RF struggles to generalize across complex, high-dimensional spectral variations induced by differences in coalification degree, mineral composition, particle size, and measurement environments.

With the accumulation of more than 20,000 coal samples collected from multiple sources, the primary challenge has shifted from data scarcity to effective utilization of large-scale, high-diversity datasets. Traditional models, which rely heavily on localized statistical relationships, tend to capture only limited patterns and exhibit degraded performance when extrapolating across broad compositional domains. As a result, their predictive accuracy and robustness decline when applied to heterogeneous coal samples encountered in real-world industrial settings.

Deep learning provides a promising alternative due to its strong representation learning capability and scalability with data volume. When trained on sufficiently large datasets, deep neural networks can model complex nonlinear relationships and learn invariant features that are difficult to capture with conventional approaches. This makes them particularly suitable for constructing a unified, general-purpose calorific value prediction model applicable to coals of different ranks, sources, and compositional characteristics. Although several previous studies have attempted to apply deep learning techniques to coal calorific value prediction [11,13,23], their performance has often been constrained by limited dataset sizes. Under small-sample conditions, nonlinear neural networks are prone to overfitting and may fail to fully exploit their representational capacity. In some cases, their testing performance has even been inferior to that of simpler linear models [24], highlighting the critical importance of large-scale data for realizing the advantages of deep architectures.

In this context, the objective of this study is to develop a large-scale, generalizable coal calorific value prediction framework based on NIRS-XRF analysis. Rather than focusing on small-sample learning, we emphasize leveraging data diversity and volume to enhance model robustness and universality. To this end, we propose a multi-perspective fusion framework built upon Transformer architectures. The model employs a dual-pathway Transformer encoder to independently extract high-level representations from NIRS and XRF spectra, enabling effective modeling of long-range dependencies and cross-band interactions inherent in large, complex datasets.

Furthermore, to improve feature diversity and reduce bias introduced by individual pre-processing methods, we adopt a multi-perspective ensemble strategy. Multiple models trained on spectra processed with different pre-processing techniques are integrated through a Multi-View Transformer fusion module. This design allows the model to aggregate complementary information from different spectral perspectives, resulting in a robust and accurate prediction model capable of generalizing across a wide range of coal types and application scenarios.

The main contributions of this work are summarized as follows:

- We propose a dual-pathway Transformer architecture for fusing NIRS and XRF spectra. This design extracts features from two different types of spectrum and integrates them effectively, ensuring comprehensive information utilization from both data sources.
- We introduce a Masked Autoencoder pre-training strategy. By learning to reconstruct masked spectral parts based on internal signal correlations, this method allows us to utilize data with unreliable labels. This significantly improves the model’s ability to extract features and handle noise.
- We develop a multi-view fusion framework that combines models trained with different pre-processing methods. This strategy reduces the bias of using a single method and achieves superior prediction accuracy and robustness for coal calorific value.

## 2. NIRS-XRF Fusion Spectroscopy System Setup

### 2.1. NIRS-XRF Bi-Spectrometer

**Figure 1:**
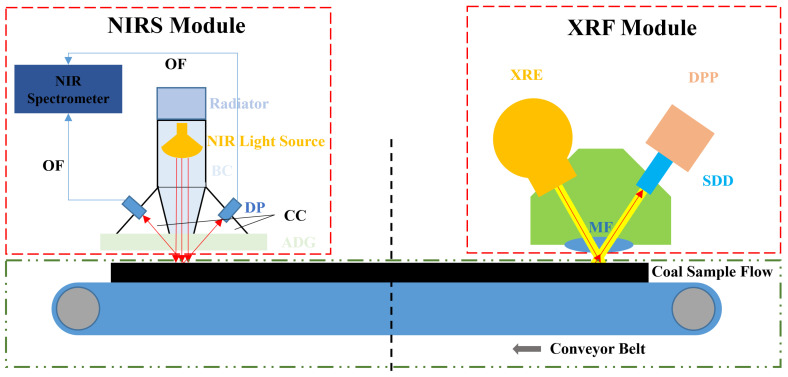
Accurate coal calorific value analyzer based on a NIRS-XRF bi-spectrometer. “OF” represents optical fiber. “DP” is the detection probe. “BC” is the beam chamber. “CC” denotes the collection chamber. “ADG” represents anti-dust glass. “XRE” denotes the X-ray emitter. “DPP” represents the digital pulse processor. “SDD” denotes the silicon drift detector. “MF” represents mylar film. The red arrows indicate the transmission path of the near-infrared light and X-ray beam, and the grey arrow indicates the movement direction of the conveyor belt and coal sample flow.

**Figure 2:**
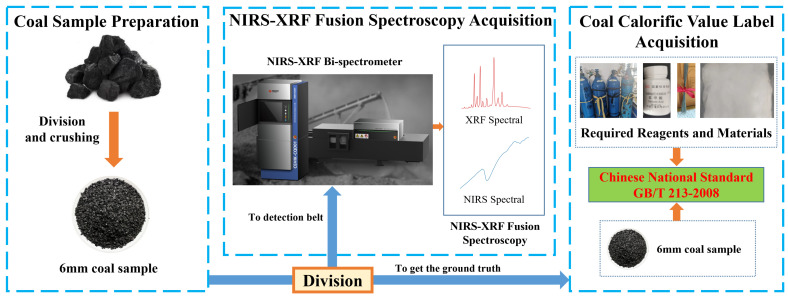
The pipeline of data acquisition. Raw coal from the transport belt is sampled and processed through multi-stage division and crushing to a particle size of 6 mm. On the conveyor belt, the coal stream is shaped by a pressing roller and sequentially passes beneath the XRF and NIR spectrometers for data collection. Finally, we use the Chinese National Standard GB/T 213-2008 [5] to obtain the ground truth of coal calorific value. The upper-right panel shows the required reagents and materials for the measurement: from left to right, oxygen, benzoic acid, ignition wire, and acid-washed asbestos.

Figure 1 presents the schematic diagram of the detector, while the central part of Figure 2 displays its physical photograph. The acquisition workflow of NIRS-XRF fusion spectroscopy, illustrated in Figure 1, consists of several main stages. First, the coal sample is placed under the NIRS module so that the NIRS spectrum can be collected. Next, the motor driven by the PLC turns the coal sample stage, moving the sample under the XRF module, where the XRF spectrum is then obtained. At the end, the PLC-controlled motor rotates the stage once more so that the coal sample can be taken out of the system. The overall dimensions of the NIRS-XRF bi-spectrometer are 1200mm×1200mm×1800mm.

The NIRS-XRF bi-spectrometer is built from six principal parts: the NIRS module, the XRF module, the rotating coal sample delivery stage, the control module, the hydrogen production module, and the operation software. The NIRS module and XRF module are described in detail below.

#### 2.1.1. NIRS Module

As shown in Figure 1, the optical structure of the NIRS module includes the following major components: a near-infrared light source, a near-infrared spectrometer, detection probes, optical fiber, a radiator, a beam chamber, a collection chamber, and anti-dust glass. A halogen lamp serves as the near-infrared light source; it covers a wavelength range of 360–2500 nm and, under low power mode, has a color temperature of 2700 K together with a service life of more than 13,000 h. The spectrometer adopts the Fourier transform design and integrates a miniature Michelson spectral interferometer with its control circuit. Its effective working band spans 1100–2500 nm, with a signal-to-noise ratio reaching 10,000:1, a spectral resolution of 5.7 nm, and spectral reproducibility within ±0.5 nm.

A total of 15 centrally symmetric detection probes are arranged around the central axis of the beam chamber and placed inside the collection chamber within the NIRS module. Using more probes broadens the angular range over which the near-infrared light reflected from the coal sample can be received, thereby increasing the amount, intensity, and quality of the collected reflected near-infrared light. As a result, the accuracy of the measurement is markedly improved. The role of the beam chamber is to restrict the illumination area of the near-infrared light so that it is concentrated onto the coal sample. Moreover, a light extinction structure is built into the inner wall of the chamber, which effectively removes stray light produced by reflection or scattering inside the chamber. To protect the performance and extend the service life of the near-infrared light source and detection probes, both the collection chamber and the beam chamber are sealed with anti-dust glass that preserves their integrity and proper operation.

#### 2.1.2. XRF Module

The energy-dispersive XRF (ED-XRF) technology is adopted in this module. Figure 1 shows the optical structure of the ED-XRF module, which is mainly composed of an X-ray emitter, a silicon drift detector (SDD), a digital pulse processor (DPP), and a mylar film. Inside the X-ray emitter, the required voltage between the filament and the target anode is supplied by a high voltage power supply. The filament emits electrons that are accelerated under an electric field and then strike the anode target to produce X-rays. Acting as the radiation source, these X-rays are used to excite the sample. After passing through the mylar film located at the bottom of the chamber, the X-rays reach the coal sample and stimulate secondary X-rays (X-ray fluorescence), which are subsequently captured by the SDD. The acquired signals are sent to a computer via the DPP for further analysis.

In this experiment, a 50 W/Rh target-anode X-ray emitter is employed as the excitation source. The X-ray emitter operates at a voltage of 16 kV and a current of 0.6 mA, whereas the filament is set to 1.5 V and 2.5 A. In addition, the peak time of the SDD is set to 0.1 µs.

### 2.2. Datasets

**Figure 3:**
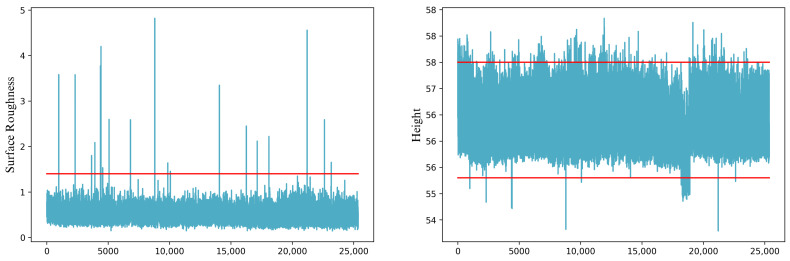
Height and surface roughness distributions of all coal samples. The x-axis denotes the sample index. The (**left**) panel shows the surface roughness distribution. The red horizontal line indicates the roughness threshold of 1.4 mm, and samples with surface roughness greater than 1.4 mm were excluded. The (**right**) panel shows the height distribution. The two red horizontal lines indicate the height thresholds of 55.3 mm and 57.5 mm, and samples with heights outside this range were excluded.

Initially, a total of 22,720 coal samples are collected and pulverized for preparation. Subsequently, detailed spectral signals are recorded using the NIRS-XRF bi-spectrometer rapid coal quality analysis system (Hangzhou Hikvision Digital Technology Co., Ltd., Hangzhou, China). The pipeline of data acquisition is shown in Figure 2. To ensure high data quality, strict protocols are established for coal sample preparation and loading. First, an automatic sampler collects raw coal samples with an initial particle size of approximately 50 to 100 mm directly from the main transport belt. These samples then undergo multi-stage division and crushing to reach a uniform particle size of 6 mm. After preparation, the processed coal is split into two paths: one portion drops into a collector for testing to get the ground truth of the calorific value of the coal samples, while the remainder is fed onto the detection conveyor belt of the NIRS-XRF bi-spectrometer system. On this belt, the coal stream is leveled by a pressing roller to provide a standardized height and surface roughness. It then sequentially passes beneath the XRF and NIR spectrometers to capture the NIRS-XRF spectra. Even with mechanical shaping, improper feeding conditions can cause variations in the surface roughness and height of the coal flow, which compromises data consistency. Therefore, specific thresholds are set for quality control. Coal samples with a height outside the range of 55.3 to 57.5 mm or a surface roughness exceeding 1.4 mm are identified as outliers and excluded during the data cleaning process. Here, the sample height refers to the height of the coal sample on the conveyor belt measured by a laser distance sensor, and surface roughness is defined as the standard deviation of the sample height recorded per second, reflecting the surface roughness of the coal sample during detection. Figure 3 shows the distribution of coal flow and the threshold. Approximately 2.06% of the initial collection is discarded, resulting in a final clean dataset including 21,785 coal samples. Data cleaning is necessary for this study because the proposed coal calorific value prediction algorithm aims to achieve high-precision prediction. Such a task requires accurate spectral equipment, standardized sample preparation, and acquisition operations. Samples with non-compliant height or surface roughness may introduce unreliable spectral signals, and these data are therefore unsuitable for model training and evaluation.

**Figure 4:**
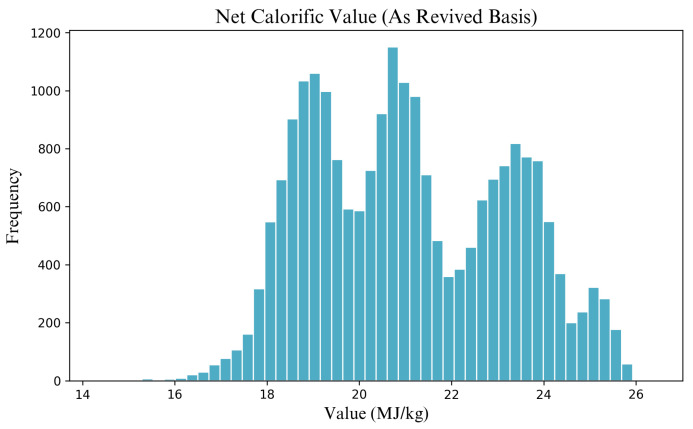
Histogram of the heat value of 21,785 coal samples after data cleaning.

Following spectral acquisition and data cleaning, the ground truth of the calorific value of the coal samples is measured in accordance with the Chinese National Standard GB/T 213-2008 [5]. As shown in Figure 4, the calorific values of the final dataset range from 14.32 MJ/kg to 26.41 MJ/kg. The coal samples in this study come from many different sources and include various coal types, resulting in a large dataset with diverse compositions. As a result, the calorific values cover a wide range and show an uneven distribution. This diversity makes the relationship between spectral signals and calorific value more complex and difficult to model using traditional methods such as Partial Least Squares (PLS) and other simple machine learning approaches.

## 3. Methodology

The analysis of spectra in large-scale coal quality datasets is challenging due to complex matrix effects and the mutual interference between elemental peaks. Furthermore, accurate prediction requires the effective fusion of dual-source data: Near-Infrared Spectroscopy (NIRS) and X-ray Fluorescence (XRF). To address these challenges, we propose a novel deep learning framework.

This section details the proposed methodology in three parts: first, the foundational dual-spectrum fusion Transformer architecture (DFFormer); second, the self-supervised pre-training strategy based on Masked Autoencoders; and third, the final Multi-View Transformer (MVFormer) that integrates multiple pre-processing views for more robust and reliable prediction performance.

### 3.1. Dual-Spectrum Fusion Transformer (DFFormer)

**Figure 5:**
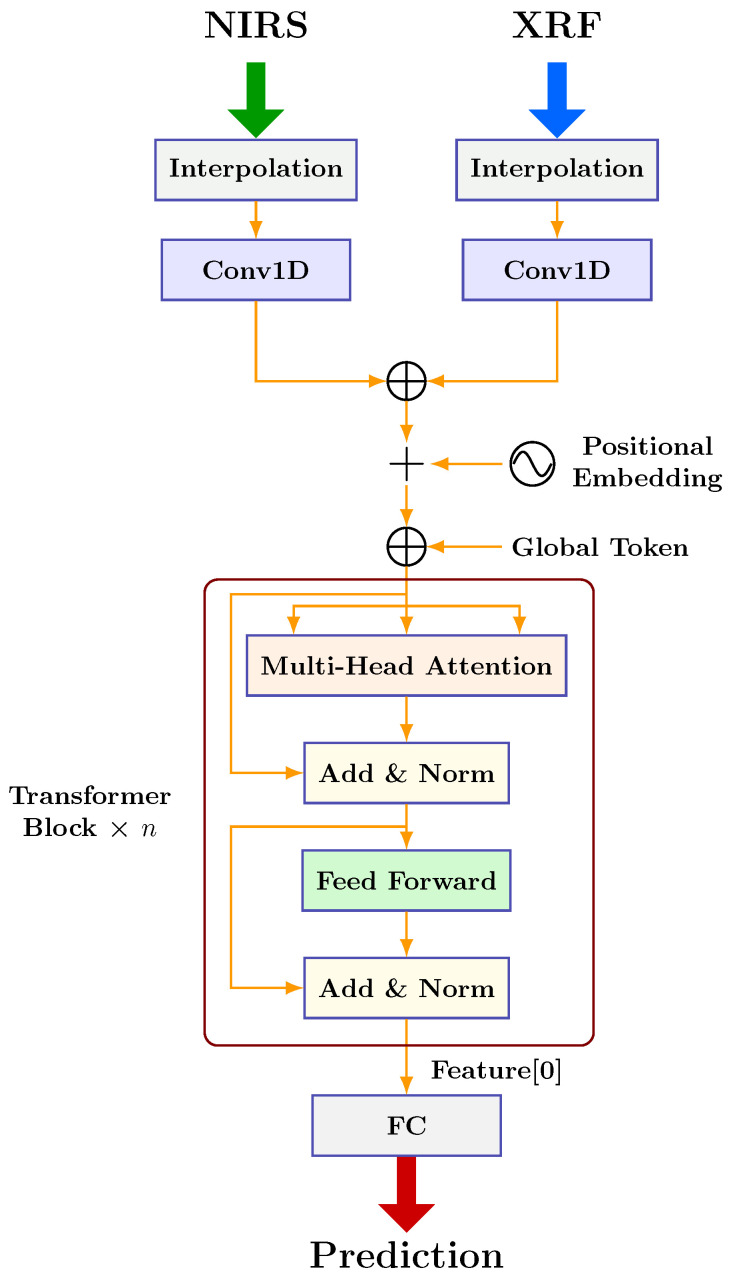
The architecture of the Dual-spectrum Fusion Transformer (DFFormer). NIRS and XRF spectra are interpolated and separately encoded by one-dimensional convolutional layers (Conv1D), then fused with positional embeddings and a global token. The fused sequence is processed by stacked Transformer blocks, and the global-token feature is fed into a fully connected layer for prediction.

Transformers has been widely adopted in Natural Language Processing (NLP) [25] and Computer Vision (CV) [26] for its ability to model long-range dependencies. This mechanism is also well-suited for spectral analysis, as spectra can be treated as sequential data. Inspired by the Vision Transformer (ViT) [27], we adapt this architecture to treat spectral data as sequences of patches. However, standard Transformer components cannot be applied directly to dual-spectral data because NIRS and XRF signals have different lengths and structural characteristics. Simply connecting them would create unaligned features, making extraction difficult. To solve this, we design the Dual-spectrum Fusion Transformer (DFFormer), as illustrated in Figure 5.

The DFFormer employs a dual-path input strategy. NIRS and XRF signals are first processed by separate, modality-specific 1D convolutional layers. These layers act as tokenizers, projecting the continuous spectral signals into feature embeddings with consistent dimensions. Assuming a segmentation strategy that divides each spectrum into distinct patches, the feature tokens from both modalities are concatenated to form a unified sequence. To preserve spectral order, learnable positional encodings are added to these tokens. Crucially, a learnable Global Token is appended to the sequence to aggregate global information for the regression task. The core of the model consists of stacked Transformer Blocks performing multi-head self-attention. This mechanism allows the model to capture long-range dependencies and facilitates robust feature interaction between the two spectra. Finally, the Global Token is extracted, normalized, and passed through a Fully Connected head to generate the prediction.

### 3.2. Masked Autoencoder Pre-Training

Masked Autoencoder method provides a powerful framework for unsupervised learning, and has achieved significant success in computer vision [28]. We adapt this self-supervised paradigm to spectral analysis based on the specific characteristics of our data. In energy spectra, elemental peaks are subject to matrix effects, resulting in interactions where information diffuses across adjacent peaks. Furthermore, NIRS and XRF signals contain complementary information. Consequently, even if parts of the signal are masked, the missing information can be reconstructed using the context from other energy bands and the complementary spectrum.

In industrial scenarios, data quality is frequently compromised. Factors such as improper sample preservation, analysis deviations, inclusion of foreign materials, non-standard signal acquisition, dust contamination, or weakening light source intensity can lead to instances where the collected data are inaccurate or inconsistent with the true values. We refer to these compromised samples as “dirty data.” While such data are often discarded in supervised learning, they still retain intrinsic spectral correlations. To utilize this information, we employ a Masked Autoencoder strategy designed for dual-spectral data. This approach leverages both high-quality and dirty data to reconstruct masked NIRS-XRF signals, thereby enhancing the network’s feature extraction capability and robustness against disturbances.

**Figure 6:**
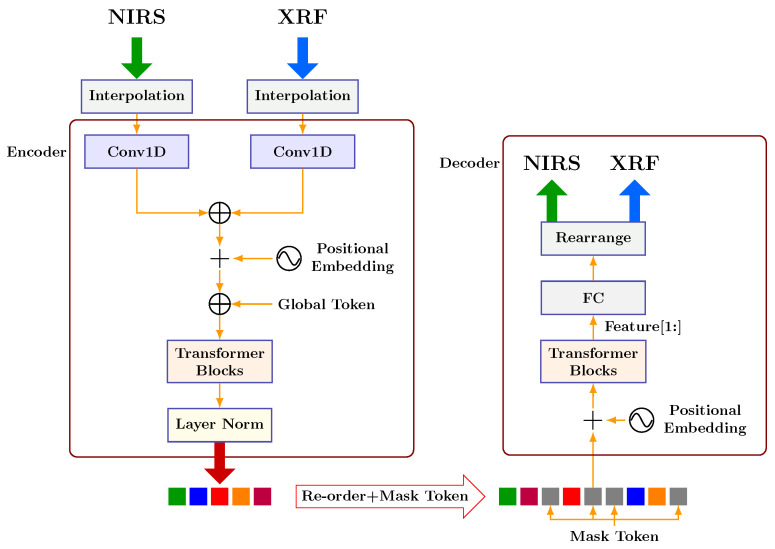
The schematic of Masked Autoencoder Pre-training. NIRS and XRF spectra are first interpolated and separately encoded by one-dimensional convolutional layers (Conv1D), then fused with positional embeddings and a global token. The encoded tokens are reordered, partially masked, and passed through a Transformer decoder to reconstruct the original NIRS and XRF spectra.

As illustrated in Figure 6, the overall pre-training architecture consists of an asymmetric Encoder–Decoder design. The Encoder structure is identical to the supervised model shown in Figure 5 but excludes the final fully connected (FC) classification layer. After pre-training, only the Encoder parameters are reused for subsequent tasks. The specific workflow is as follows:

1. Masking and Shuffling: The input NIRS and XRF signals are processed into feature tokens. A portion of these tokens is randomly masked to hide information. To prevent the network from relying solely on fixed positions, the sequence of the remaining visible tokens is shuffled before being input into the network.
2. Sparse Encoding: Only the visible tokens (including the Global Token) are fed into the Encoder. This allows the Encoder to process a shorter sequence, extracting high-level latent representations from the partial observations.
3. Token Restoration and Reordering: Before entering the Decoder, the sequence must be restored. The output features from the Encoder are re-sorted to their original order. We introduce a learnable “Mask Token” to fill the positions of the missing data. The Global Token is maintained at the first position to preserve the aggregated context.
4. Reconstruction: The full sequence (reordered features plus Mask Tokens) is combined with positional encoding and fed into the Decoder blocks. Finally, the Global Token is removed, and the remaining tokens pass through a fully connected layer to reconstruct the original NIRS and XRF signals.

The model is optimized by minimizing the reconstruction loss between the predicted and original signals. The loss function is defined as:

(1) $$L_{\mathrm{MAE}}=\frac{\sum {\left(X_{\mathrm{NIRS}}-\hat{X_{\mathrm{NIRS}}}\right)}^{2}m_{\mathrm{NIRS}}+\sum {\left(X_{\mathrm{XRF}}-\hat{X_{\mathrm{XRF}}}\right)}^{2}m_{\mathrm{XRF}}}{\sum m_{\mathrm{NIRS}}+\sum m_{\mathrm{XRF}}}$$

where *X* and $\hat{X}$ denote the original interpolated signal data and the reconstructed data, respectively; and *m* denotes the binary masks (1 for masked, 0 for visible) for the NIRS and XRF signals. This pretext task allows the model to learn high-level abstractions of the coal spectra structure, significantly benefiting the downstream regression task.

### 3.3. Multi-View Transformer (MVFormer)

**Figure 7:**
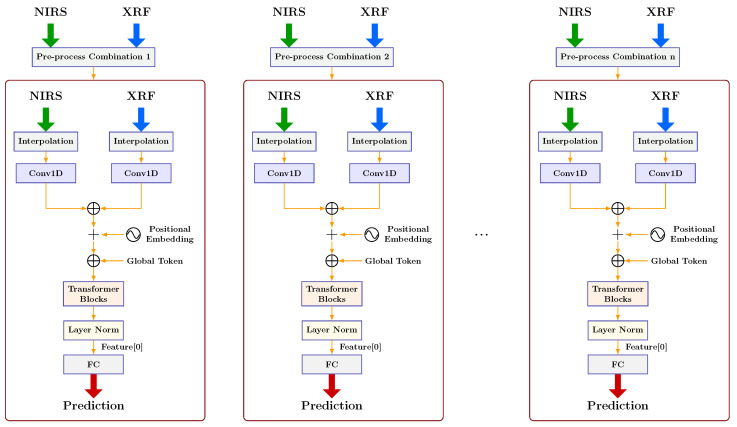
Independent pre-training of multiple sub-models with different pre-process combinations of NIRS and XRF signals. Each sub-model adopts a unique combination of preprocessing methods while sharing the same DFFormer architecture for independent training. The ellipsis (…) indicates that an arbitrary number of sub-models can be constructed using different preprocessing combinations.

**Figure 8:**
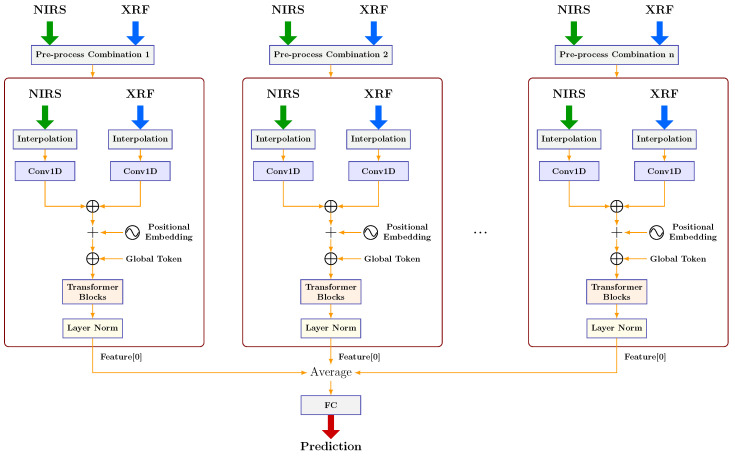
MVFormer framework with feature fusion of multiple sub-models. Global features (Feature[0]) extracted by independently trained DFFormer sub-models with different preprocessing combinations are fused by average pooling and then passed to a fully connected (FC) layer for final prediction. The ellipsis (…) indicates that the framework can include an arbitrary number of sub-models.

Signal pre-processing is essential to reduce noise, but different methods preserve different aspects of the spectral information. Consequently, a single model trained on one type of pre-processed data may converge to a local optimum, failing to capture the full complexity of the underlying signal. To capitalize on the diversity of spectral features and improve model robustness, we propose the Multi-View Transformer (MVFormer). The core idea of MVFormer is to treat different pre-processing strategies as distinct “views” of the data. By integrating sub-models trained on these diverse views, the framework leverages complementary information that a single view might miss. As shown in Figure 7 and Figure 8, the MVFormer operates in two stages:

- Independent Pre-training: We instantiate multiple sub-models, where each corresponds to a specific pre-processing view. These models are independently pre-trained using Masked Autoencoder strategy described in Section 3.2. This approach ensures that each sub-model focuses on learning the unique feature representations specific to its assigned view.
- Feature Fusion Training: The pre-trained sub-models are integrated into the unified MVFormer framework. To ensure stability and efficiency during this phase, the weights of the sub-models are frozen, and only the subsequent fusion layers are updated during the MVFormer training.

In the fusion stage, the global feature representations (Global Tokens) from all sub-models are aggregated. We utilize a global feature fusion strategy where these features are concatenated and passed through a final Multilayer Perceptron (MLP) head. The MLP employs ReLU activation and Dropout regularization to prevent overfitting. This multi-view design ensures that MVFormer comprehensively effectively synthesizes complementary information from heterogeneous sources, yielding a robust solution for high-precision coal quality prediction.

## 4. Experiments

### 4.1. Experiment Settings

To determine the optimal model configuration, we conducted a comprehensive series of ablation studies. These experiments investigated the impact of varying data scales, the necessity of data cleaning, network architectures, and hyperparameter settings. During this model optimization phase, we employed 10-fold random cross-validation [29] to ensure the reliability and robustness of our comparative analysis. The dataset is randomly split into 10 equal parts. In each fold of the cross-validation process, the model is trained on nine parts of the dataset and the remaining one part is used for testing. The testing part changes in each iteration to ensure that the model generalizes well to new data.

Upon identifying the optimal configuration, the final performance of the model is evaluated using a rigorous data partitioning strategy. The SPXY method was applied to the cleaned dataset to select a representative subset comprising 90% of the remaining samples for training [30], with the remaining 10% reserved as the independent test set. The coal samples in our dataset exhibit substantial diversity in terms of geographical origin, calorific value, and collection time. Each sample was independently collected and subjected to separate laboratory analysis and measurement, ensuring that the samples are mutually independent. All experiments were conducted on a workstation equipped with an Intel Xeon Gold 6132 processor (Intel Co., Santa Clara, CA, USA) and an NVIDIA Tesla V100 GPU (NVIDIA Co., Santa Clara, CA, USA). The experimental environment was built using Python (v.3.8.5). For deep learning experiments, the models were implemented using PyTorch (v.1.11.0) with CUDA (v.11.3). Key machine learning and data processing libraries included Pandas (v.1.3.4), and NumPy (v.1.19.4). All model training, validation, and testing procedures were performed under the same hardware and software environment to ensure fair comparison and reproducibility.

### 4.2. Evaluation Metrics

The algorithm’s accuracy is assessed using various evaluation metrics on the calibration and test sets, including the mean absolute error (MAE), root mean square error (RMSE), mean absolute relative error (ARE), correlation coefficient ($R^{2}$), and mean square error (MSE). The formulations for these metrics are provided as follows:

(2) $$\mathrm{MAE}=\frac{1}{n}\sum_{i=1}^{n}\left|y_{i}-{\hat{y}}_{i}\right|,$$

(3) $$\mathrm{RMSE}=\sqrt{\frac{\sum_{i=1}^{n}{\left(y_{i}-{\hat{y}}_{i}\right)}^{2}}{n}},$$

(4) $$\mathrm{ARE}=\frac{1}{n}\sum_{i=1}^{n}\frac{|y_{i}-{\hat{y}}_{i}|}{y_{i}},$$

(5) $$R^{2}=1-\frac{\sum_{i=1}^{n}{\left(y_{i}-{\hat{y}}_{i}\right)}^{2}}{\sum_{i=1}^{n}{\left(y_{i}-\bar{y}\right)}^{2}},$$

(6) $$\mathrm{MSE}=\frac{1}{n}\sum_{i=1}^{n}{\left(y_{i}-{\hat{y}}_{i}\right)}^{2},$$

where *y* and $\hat{y}$ represent the ground truth and predicted calorific values, respectively. Here, *n* denotes the number of samples in the datasets, while $\bar{y}$ is the average of the ground-truth calorific values in the datasets.

### 4.3. Implementation Details

#### 4.3.1. Data Pre-Processing

Raw spectral data collected from Near-Infrared Spectroscopy (NIRS) and X-ray Fluorescence (XRF) sensors are often affected by noise and interference. During the data collection process, the samples are easily influenced by environmental and instrumental factors. Common examples include light scattering caused by poor sealing of the dark box, stray light from outside sources, and standard instrument noise. These interfering signals can seriously lower the spectral quality and distort the data, which directly reduces the prediction accuracy of the models. Therefore, data pre-processing is necessary in this study.

To reduce noise in the raw NIRS and XRF spectra, Savitzky–Golay smoothing is applied [31]. Before modeling, specific spectral regions are chosen to keep only the useful bands: the analysis ranges are limited to 1600–2400 nm for NIRS and 1.4–9.9 keV for XRF. However, data processed by different methods often contain different information. By using data from various pre-processing methods to train the network, the model can access more information and potentially learn better features. Consequently, for NIRS spectra, four different pre-processing methods are employed [32]:

- Area Normalization (AN): Scales the spectrum to unit area:
  (7) $$X_{\mathrm{AN}}^{\mathrm{NIRS}}=\frac{X^{\mathrm{NIRS}}}{\sum_{i=1}^{D_{\mathrm{NIRS}}}x_{i}^{\mathrm{NIRS}}}$$
- First Derivative (FD): Performs first-order differentiation on the raw signal:
  (8) $$X_{\mathrm{FD}}^{\mathrm{NIRS}}=\frac{d}{d\lambda }X^{\mathrm{NIRS}}$$
- Area Normalization followed by First Derivative (ANFD): Applies the first derivative calculation after area normalization:
  (9) $$X_{\mathrm{ANFD}}^{\mathrm{NIRS}}=\frac{d}{d\lambda }\left(\frac{X^{\mathrm{NIRS}}}{\sum_{i=1}^{D_{\mathrm{NIRS}}}x_{i}^{\mathrm{NIRS}}}\right)$$
- Standard Normal Variate (SNV): Centers and scales each spectrum based on its mean and standard deviation:
  (10) $$X_{\mathrm{SNV}}^{\mathrm{NIRS}}=\frac{X^{\mathrm{NIRS}}-\mu_{\mathrm{NIRS}}}{\sigma_{\mathrm{NIRS}}}$$
  where $\mu_{\mathrm{NIRS}}$ and $\sigma_{\mathrm{NIRS}}$ are the mean and standard deviation of the NIRS spectrum, respectively.

For XRF spectra, two pre-processing methods are implemented:

- Full Spectrum (FS): Utilizes the complete XRF signal after smoothing and range selection.
- Peak Area (PA): Computes the area under each X-ray characteristic peak to form a feature sequence.

The combination of four NIRS pre-processing methods with two XRF pre-processing methods yields eight distinct pre-processing combinations. Each combination represents a unique data representation that captures different aspects of the spectral information These eight pre-processing variants correspond to eight individual Transformer sub-models in our ensemble framework, allowing the model to leverage complementary information from diverse spectral representations.

Following individual pre-processing of NIRS and XRF spectra, the combined spectral features are standardized using the mean and standard deviation of the training dataset.

#### 4.3.2. Ablation Experiment

To determine the optimal hyperparameters for the proposed model, a series of ablation experiments are conducted. We investigated the impact of embedding dimensions, the number of encoder layers, the number of attention heads, the size of the training set, and data cleaning on the model’s performance. All experiments are evaluated based on the coefficient of $R^{2}$, MAE, MSE, RMSE, and ARE introduced in Section 4.2. For the ablation studies of the embedding dimension, the number of encoder layers, the number of attention heads, the mask ratio of Masked Autoencoders, and the batch size of Masked Autoencoders, 10-fold random cross-validation is adopted. It is used to evaluate the stability and robustness of model performance under different hyperparameter settings. The average performance and standard deviation (mean ± std) across the 10 folds are reported as the experimental results.

**Effect of Embedding Dimension:** The embedding dimension determines the capacity of the model to represent spectral features in the latent space. As shown in Table 1, we tested dimensions of 32, 64, 128, and 256. The model achieved the best performance with an embedding dimension of 128, yielding the highest $R^{2}$ of 0.9843 and the lowest RMSE of 0.2668 MJ/kg. Lower dimensions (32 and 64) likely restricted the feature representation capability, while a dimension of 256 led to a slight degradation in performance, potentially due to overfitting on the limited dataset.

**Effect of Number of Layers:** The depth of the Transformer encoder is varied from one to five layers. Table 2 indicates that a depth of four layers provides the optimal balance between model complexity and generalization, achieving an $R^{2}$ of 0.9842. Shallower networks (1–3 layers) underperformed, suggesting insufficient capacity to capture complex non-linear relationships in the NIRS-XRF fusion spectra. Conversely, increasing the depth to five layers did not yield further improvements and slightly increased the error, likely due to the vanishing gradient problem or optimization difficulties associated with deeper networks on this dataset size.

**Effect of Attention Heads:** The multi-head attention mechanism allows the model to focus on different subspaces of the spectral data simultaneously. As presented in Table 3, increasing the number of heads from two to eight resulted in consistent improvements. The configuration with eight heads achieved the best performance ($R^{2}=0.9846$, RMSE = 0.264 MJ/kg), demonstrating that a finer granularity in attention distribution helps in extracting more robust spectral features.

**Effect of Mask Ratio of Masked Autoencoders:** In the Masked Autoencoder framework, the mask ratio is a crucial hyperparameter that determines the difficulty of the self-supervised reconstruction task. A ratio that is too low may result in a trivial task, while a ratio that is too high may lead to information loss. As shown in Table 4, we experimented with mask ratios of 0.25, 0.50, and 0.75. The model achieved optimal performance at a mask ratio of 0.50, reaching an $R^{2}$ of 0.9850 and the lowest RMSE of 0.2611 MJ/kg. This suggests that masking half of the spectral tokens forces the model to learn robust contextual representations without hindering its ability to reconstruct the signal.

**Effect of Batch Size of Masked Autoencoders:** The batch size used during Masked Autoencoder pre-training impacts the stability of gradient estimation and the model’s convergence. We compared the performance differences between batch sizes of 5000 and 10,000, as detailed in Table 5. Increasing the batch size to 10,000 yielded a slight but noticeable improvement, boosting the $R^{2}$ to 0.9850 and reducing the MAE to 0.1987 MJ/kg. The larger batch size likely facilitated more stable optimization dynamics during the representation learning phase, resulting in better generalization on the downstream regression task.

**Effect of Training Set Size:** The scale of the dataset is critical for deep learning models. Table 6 illustrates a clear positive correlation between dataset size and model performance. Reducing the training data to 25% drastically lowered the $R^{2}$ to 0.969 and increased the RMSE to 0.4208 MJ/kg. This confirms that collecting a large-scale NIRS-XRF dataset is justifiable and necessary for high-precision modeling. For the ablation study on training set size, the SPXY method is used to select 90% of the samples as the training set, while the remaining 10% are used as the test set. To evaluate the influence of training data size, the number of training samples selected by SPXY is further reduced to 75%, 50%, and 25% of the original 90% training set, while the test set remains unchanged. Each experiment was repeated 10 times with different random seeds, and the performance is reported as the (mean ± std) over these ten runs. This setting allows the model performance to be compared under different amounts of training data using the same test set, which makes the effect of training set size more direct and clear.

**Effect of Data Cleaning:** Table 7 presents the effect of the data cleaning protocol described in Section 2.2. The protocol aims to reduce errors caused by variations in coal sample height and surface roughness. As shown in the table, the model trained on cleaned data achieves better performance than that trained on uncleaned data. These results suggest that high-quality spectral inputs are important for model convergence and prediction accuracy, and that removing samples with obvious physical irregularities can improve the reliability of the prediction results. For the ablation study on data cleaning, the experiments are conducted on both the cleaned and uncleaned datasets. In each case, the SPXY method is used to select 90% of the samples for training on the cleaned and uncleaned datasets, and the remaining 10% are used for testing. Each experiment was repeated 10 times with different random seeds, and the performance is reported as the (mean ± std) over these ten runs.

#### 4.3.3. Training Details

**Figure 9:**
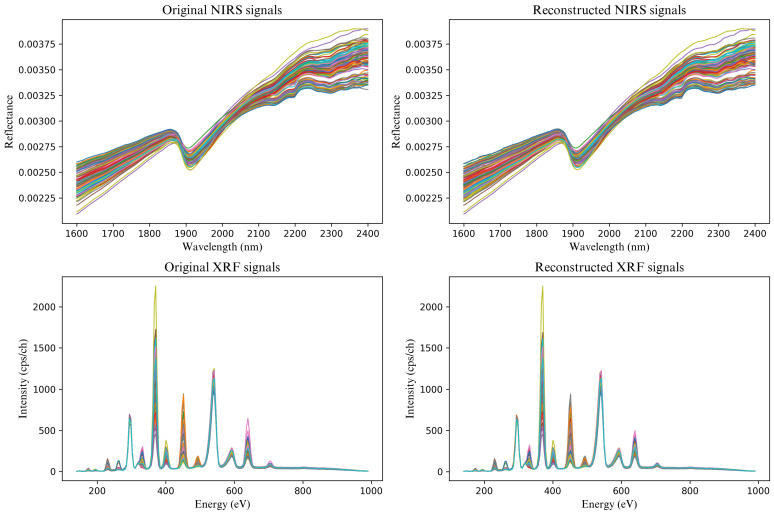
The comparison between original NIRS/XRF signals and signals reconstructed by the Masked Autoencoders. Twenty spectra were randomly selected for visualization. The (**left**) panels show the original NIRS and XRF signals, while the (**right**) panels present the corresponding reconstructed signals obtained by the Masked Autoencoders.

For the Masked Autoencoder pre-training, the network parameters are optimized using the Adam optimizer for a total of 10,000 epochs. The learning rate is set to $1\times 10^{-3}$ with a weight decay rate of $1\times 10^{-4}$. The network parameters of the eight Transformer sub-models with Masked Autoencoders are optimized using the Adam optimizer for a total of 10,000 epochs. The learning rate is set to $1\times 10^{-4}$ with a weight decay rate of $1\times 10^{-4}$. Figure 9 shows some examples of the comparison between original NIRS-XRF signal and the signal reconstructed by the Masked Autoencoders, demonstrating that the Masked Autoencoders can effectively and accurately reconstruct the spectral signals.

**Figure 10:**
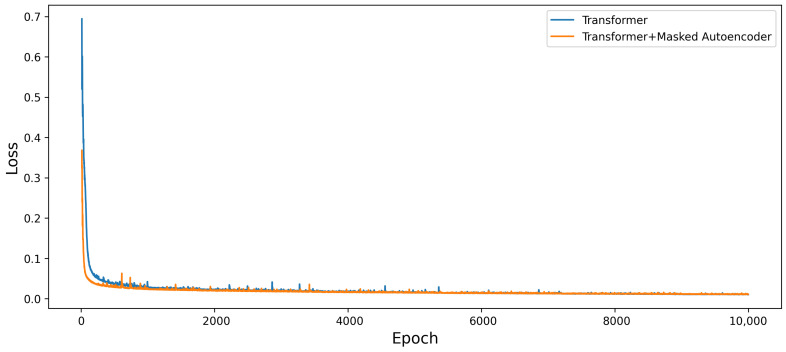
The Comparison of the training loss curves for the Transformer with and without Masked Autoencoder pre-training over 10,000 epochs.

As a baseline, eight Transformer models without Masked Autoencoders are trained using the Adam optimizer for 10,000 epochs, with a learning rate of $1\times 10^{-3}$ and a weight decay rate of $1\times 10^{-4}$. Figure 10 illustrates the loss curves of the Transformer model with and without Masked Autoencoders, the model with Masked Autoencoders converges more rapidly during training.

The MVFormer serves as an ensemble framework integrating eight MAE-based Transformer sub-models, each utilizing different pre-processing techniques. During the training of MVFormer, the parameters of these sub-models are frozen. The mean of the features extracted by different sub-models is fed into a Multi-Layer Perceptron (MLP) for final prediction. To further prevent overfitting, the MLP applies a dropout rate of 0.1. The MVFormer is trained for 500 epochs using the Adam optimizer with a learning rate of $1\times 10^{-3}$ and a weight decay rate of $1\times 10^{-4}$. For comparison, a MVFormer without Masked Autoencoders is trained using the Adam optimizer for 500 epochs, with a learning rate of $1\times 10^{-4}$ and a weight decay rate of $1\times 10^{-4}$, based on eight sub-models without Masked Autoencoders.

## 5. Results

Before conducting a comprehensive model comparison, we evaluated various data processing strategies to establish an optimal baseline. As presented in Table 8, the AN-FS method demonstrated superior performance across all metrics. Specifically, this method yielded the lowest prediction errors and the highest $R^{2}$. Consequently, in the subsequent comparative analysis, the performance of individual sub-models is represented by the sub-model using the AN-FS pre-process method.

To ensure a reliable performance evaluation, we partitioned the cleaned dataset using the SPXY method, selecting 90% of the samples as the training set and the remaining 10% as the test set. Each experiment was repeated 10 times with different random seeds, and the performance is reported as the (mean ± standard deviation) over these ten runs. Since the fitting of the PLS model is deterministic, no standard deviation is listed for it. Table 9 summarizes the quantitative performance of different regression models. Here, “Transformer” and “MAE + Transformer” represent the single-view sub-models using AN-FS pre-processing, which was specifically selected as it yielded the best performance in different single-view models. Conversely, “MVFormer” and “MAE + MVFormer” refer to the models that adopt MVFormer network, which fuse features from eight different sub-models. The traditional PLS model and a three-layer MLP network [33] are employed as baseline mothods for comparision. In addition, we introduce Light Gradient Boosting Machine (LightGBM) [34], eXtreme Gradient Boosting (XGBoost) [35], and Residual Neural Network (ResNet) [36] as further baselines to provide a more comprehensive evaluation.

The PLS model was configured with the number of components set to 75. The XGBoost model adopted 100 estimators, a maximum tree depth of 6, a learning rate of 0.1, a subsample ratio of 0.8, and a column subsample ratio per tree of 0.8. The LightGBM model used 100 estimators, a learning rate of 0.1, 31 leaves per tree, a subsample ratio of 0.8, a column subsample ratio per tree of 0, and a minimum of 20 samples per leaf, with no depth limit imposed.

Adopting the same dual-pathway structure as the Transformer, the MLP processes the NIRS and XRF features through two separate single-layer fully connected networks, each mapping the input to 128 dimensions followed by a Tanh activation. The two outputs are then concatenated into a 256-dimensional vector, compressed back to 128 dimensions through a fully connected layer with another Tanh activation, and finally mapped to a one-dimensional prediction via the output layer.

For the ResNet model, the concatenated NIRS and XRF spectra, forming a one-dimensional sequence of length 400, are reshaped into a 20×20 two-dimensional feature map to enable the application of 2D convolutions. It is first processed by an initial convolutional layer with 64 output channels, a 3×3 kernel, and a Tanh activation. The features then pass through four residual layers, each containing two residual blocks, where each block consists of two 3×3 convolutional layers with skip connections and Tanh activations. The first layer maintains 64 channels with a stride of 1, while the subsequent three layers use a stride of 2 to progressively reduce the spatial resolution. Finally, global average pooling compresses the feature map into a 64-dimensional vector, which is mapped to a one-dimensional prediction through a fully connected layer.

Consequently, the proposed Transformer model significantly outperformed these conventional and simple network approaches. The results highlight the efficacy of two core components in the proposed framework: Transformer model pre-trained with MAE and the Multi-View Transformer architecture. First, the integration of Masked Autoencoders consistently enhanced model performance by enabling the network to learn robust feature representations through self-supervised pre-training. When comparing the single-view baselines, Transformer with Masked Autoencoders reduced the MAE from 0.1615 MJ/kg to 0.1471 MJ/kg and the MSE from 0.0425 (MJ/kg)^2^ to 0.0351 (MJ/kg)^2^ relative to the standard Transformer. This significant reduction in prediction error validates the utility of self-supervised pre-training in spectral regression tasks Second, the proposed MVFormer further improved predictive precision by effectively fusing complementary features from NIRS and XRF spectra. The MVFormer model surpassed the best single-view Transformer, reducing the RMSE from (0.2020±0.0094) MJ/kg to (0.1835±0.9987) MJ/kg and the ARE from (0.72±0.03)% to (0.65±0.03)%. This improvement indicates that the multi-view strategy captures global correlations that single-view models may overlook. Ultimately, the proposed MVFormer utilized with Masked Autoencoder pre-training, which synergizes self-supervised pre-training with multi-view feature fusion, achieved the best performance in this study. This model achieved an MAE of (0.1388±0.0013) MJ/kg, an MSE of (0.0301±0.0006) (MJ/kg)^2^, an RMSE of (0.1735±0.0013) MJ/kg, an ARE of (0.63±0.01)%, and an $R^{2}$ of (0.9948±0.0001). These metrics confirm that the combination of Masked Autoencoder and MVFormer significantly enhances both robustness and precision in spectral regression.

## 6. Conclusions

This study proposed a novel deep learning framework, MVFormer, which utilized Masked Autoencoders for high-precision coal quality analysis using integrated NIRS and XRF spectral data. Comparative analysis demonstrated that the proposed architecture significantly outperforms traditional PLS regression and simple MLP models. The superior performance is attributed to two primary mechanisms: the MVFormer, which maximizes information utilization from NIRS-XRF spectra through effective feature fusion, and the Masked Autoencoder pre-training strategy, which substantially enhances the model’s feature representation capability. Specifically, the MVFormer model with Masked Autoencoders achieved an MAE of (0.1388±0.0013) MJ/kg, an MSE of (0.0301±0.0006) (MJ/kg)^2^, an RMSE of (0.1735±0.0013) MJ/kg, an ARE of (0.63±0.01)%, and an $R^{2}$ of (0.9948±0.0001). These findings suggest that the proposed method offers a robust and accurate solution for rapid, non-destructive coal quality detection in industrial settings.

## Figures and Tables

**Table 1:** Performance metrics for different embedding dimensions. The best results are highlighted in bold.

Embedding Dim	*R* ^2^	MAE (MJ/kg)	MSE (MJ/kg)^2^	RMSE (MJ/kg)	ARE (%)
32	0.9835±0.0013	0.2083±0.0062	0.0746±0.0052	0.2731±0.0094	1.01±0.03
64	0.9841±0.0013	0.2045±0.0056	0.0722±0.0048	0.2687±0.0090	0.99±0.03
**128**	0.9843±0.0012	0.2029±0.0050	0.0712±0.0046	0.2668±0.0089	0.98±0.02
256	0.9831±0.0014	0.2105±0.0064	0.0766±0.0057	0.2768±0.0104	1.02±0.03

**Table 2:** Performance metrics for different layer configurations. The best results are highlighted in bold.

Num Layers	*R* ^2^	MAE (MJ/kg)	MSE (MJ/kg)^2^	RMSE (MJ/kg)	ARE (%)
1	0.9820±0.0021	0.2188±0.0097	0.0816±0.0085	0.2857±0.0145	1.05±0.04
2	0.9839±0.0012	0.2053±0.0048	0.0728±0.0046	0.2699±0.0084	0.99±0.03
3	0.9834±0.0010	0.2093±0.0057	0.0752±0.0037	0.2743±0.0068	1.01±0.02
**4 **	0.9842±0.0011	0.2035±0.0046	0.0716±0.0042	0.2677±0.0078	0.98±0.02
5	0.9836±0.0009	0.2084±0.0036	0.0741±0.0031	0.2723±0.0056	1.00±0.02

**Table 3:** Performance metrics for different attention head configurations. The best results are highlighted in bold.

Num Heads	*R* ^2^	MAE (MJ/kg)	MSE (MJ/kg)^2^	RMSE (MJ/kg)	ARE (%)
2	0.9842±0.0011	0.2035±0.0046	0.0716±0.0042	0.2677±0.0078	0.98±0.02
4	0.9844±0.0011	0.2025±0.0046	0.0705±0.0039	0.2656±0.0073	0.98±0.02
**8 **	0.9846±0.0011	0.2008±0.0045	0.0697±0.0039	0.2640±0.0074	0.97±0.02

**Table 4:** Performance metrics for different mask ratios. The best results are highlighted in bold.

Mask Ratio	*R* ^2^	MAE (MJ/kg)	MSE (MJ/kg)^2^	RMSE (MJ/kg)	ARE (%)
0.25	0.9843±0.0013	0.2042±0.0063	0.0711±0.0054	0.2667±0.0101	0.98±0.03
**0.50 **	0.9850±0.0012	0.1987±0.0053	0.0682±0.0048	0.2611±0.0092	0.96±0.03
0.75	0.9845±0.0010	0.2020±0.0058	0.0701±0.0044	0.2647±0.0084	0.98±0.03

**Table 5:** Performance metrics for different batch sizes. The best results are highlighted in bold.

Batch Size	*R* ^2^	MAE (MJ/kg)	MSE (MJ/kg)^2^	RMSE (MJ/kg)	ARE (%)
5000	0.9847±0.0011	0.1995±0.0048	0.0693±0.0043	0.2633±0.0082	0.96±0.02
**10,000 **	0.9850±0.0012	0.1987±0.0053	0.0682±0.0048	0.2611±0.0092	0.96±0.03

**Table 6:** Performance comparison between different sizes of training set. The best results are highlighted in bold.

Scale	*R* ^2^	MAE (MJ/kg)	MSE (MJ/kg)^2^	RMSE (MJ/kg)	ARE (%)
**100% **	0.9936±0.0004	0.1504±0.0042	0.0368±0.0019	0.1913±0.0047	0.69±0.02
75%	0.9916±0.0006	0.1706±0.0045	0.0478±0.0027	0.2184±0.0062	0.77±0.03
50%	0.9805±0.0009	0.2520±0.0050	0.1114±0.0047	0.3338±0.0074	1.14±0.04
25%	0.9693±0.0009	0.3175±0.0067	0.1751±0.0050	0.4184±0.0088	1.42±0.04

**Table 7:** Performance comparison between clean and uncleaned data. The best results are highlighted in bold.

Data Type	*R* ^2^	MAE (MJ/kg)	MSE (MJ/kg)^2^	RMSE (MJ/kg)	ARE (%)
**Cleaned**	0.9936±0.0004	0.1504±0.0042	0.0368±0.0019	0.1913±0.0047	0.69±0.02
Uncleaned	0.9929±0.0004	0.1561±0.0045	0.0403±0.0020	0.2008±0.0048	0.71±0.02

**Table 8:** Performance of Transformer models pre-trained with Masked Autoencoders using different pre-processing methods. The best results are highlighted in bold.

Pre-Process	*R* ^2^	MAE (MJ/kg)	MSE (MJ/kg)^2^	RMSE (MJ/kg)	ARE (%)
**AN-FS**	0.9936±0.0004	0.1504±0.0042	0.0368±0.0019	0.1913±0.0047	0.69±0.02
SNV-FS	0.9927±0.0004	0.1600±0.0043	0.0421±0.0020	0.2045±0.0047	0.73±0.02
FD-FS	0.9915±0.0004	0.1730±0.0044	0.0490±0.0025	0.2201±0.0054	0.79±0.02
ANFD-FS	0.9910±0.0005	0.1782±0.0045	0.0518±0.0026	0.2263±0.0056	0.80±0.02
AN-PA	0.9935±0.0005	0.1513±0.0062	0.0374±0.0027	0.1930±0.0067	0.69±0.03
SNV-PA	0.9919±0.0005	0.1676±0.0052	0.0466±0.0027	0.2147±0.0061	0.78±0.02
FD-PA	0.9913±0.0005	0.1758±0.0047	0.0505±0.0026	0.2234±0.0057	0.81±0.02
ANFD-PA	0.9897±0.0008	0.1909±0.0072	0.0594±0.0046	0.2418±0.0091	0.87±0.03

**Table 9:** Performance comparison of different regression models. “Transformer” and “MAE + Transformer” refer to the best-performing single-view sub-models (AN-FS) without or with Masked Autoencoders, while MVFormer variants represent the integrated Multi-View Transformer models. A PLS regression model, a LightGBM model, an XBGoost model, an MLP network model, and a ResNet model are employed for comparison. The best results are highlighted in bold.

Method	$R^{2}$	MAE (MJ/kg)	MSE (MJ/kg)^2^	RMSE (MJ/kg)	ARE (%)
PLS	0.9805	0.2603	0.1109	0.3330	1.18
LightGBM	0.9849±0.0001	0.2261±0.0012	0.855±0.0008	0.2924±0.0014	1.02±0.01
XGBoost	0.9863±0.0002	0.2159±0.0014	0.0781±0.0012	0.2794±0.0021	0.98±0.01
ResNet	0.9878±0.0012	0.2111±0.0117	0.0696±0.0068	0.2635±0.0128	0.96±0.05
MLP	0.9911±0.0001	0.1797±0.0015	0.0507±0.0007	0.2251±0.0016	0.81±0.01
Transformer	0.9928±0.0007	0.1597±0.0079	0.0409±0.0038	0.2020±0.0094	0.72±0.03
MAE + Transformer	0.9936±0.0008	0.1504±0.0095	0.0368±0.0045	0.1913±0.0109	0.69±0.04
MVFormer	0.9940±0.0006	0.1450±0.0066	0.0338±0.0034	0.1835±0.0087	0.65±0.03
**MAE + MVFormer**	0.9948±0.0001	0.1388±0.0013	0.0301±0.0006	0.1735±0.0013	0.63±0.01

## Data Availability

The raw data supporting the conclusions of this article are not publicly available at this time but may be obtained from the author upon reasonable request at tianzhefei@hikvision.com.
